# The significance of m6A RNA methylation regulators in predicting the prognosis and clinical course of HBV-related hepatocellular carcinoma

**DOI:** 10.1186/s10020-020-00185-z

**Published:** 2020-06-17

**Authors:** Qiongxuan Fang, Hongsong Chen

**Affiliations:** Peking University People’s Hospital, Peking University Hepatology Institute and Beijing Key Laboratory of Hepatitis C and Immunotherapy for Liver Diseases, Beijing, 100044 China

**Keywords:** Gene signature, Hepatitis B virus, Hepatocellular carcinoma, N6-methyladenosine (m6A), Prognosis

## Abstract

**Background:**

Hepatocarcinogenesis is reportedly correlated with abnormal m6A modifications; however, it is unknown whether m6A RNA methylation regulators facilitate the occurrence of hepatitis B virus (HBV)-related hepatocellular carcinoma (HCC). Thus, we constructed an m6A-related model that may enhance HBV-related HCC prognosis.

**Methods:**

Gene signatures of *HNRNPA2B1* and *RBM15* were generated by univariate and Lasso Cox regression analyses using the gene set and clinical information from The Cancer Genome Atlas (TCGA) database. High-risk and low-risk groups were confirmed based on the gene signature model. Furthermore, we validated the predictive roles of the two genes for overall survival (OS) in the GSE14520 dataset. The relative expression of 22 paired mRNAs was measured using quantitative real-time polymerase chain reaction (qRT-PCR) analysis to determine whether the two genes had a predictive role in our Guilin cohort.

**Results:**

The differences in OS between the high-risk and low-risk groups were statistically significant in the TCGA (*p* = 0.003) and GSE14520 (*p* = 0.045) datasets, but not in the Guilin cohort, owing to differences in clinical information among the three cohorts (mainly the TNM stage and survival state). Stratified analysis of TNM stages showed that the two-gene signature acted as a prognostic indicator of HBV-related HCC patients in the early TNM stage; both TCGA and GSE14520 cohorts showed statistical significance. Moreover, multivariate Cox regression analysis indicated that the two-gene signature was an independent factor for predicting prognosis (HR = 1.087, 95% CI: 1.007–1.172). Correlation analysis between the gene signature and clinical features revealed that the risk stratification was significantly correlated with grade and survival state. Finally, Gene Set Enrichment Analysis (GSEA) revealed that the KEGG pathways associated with the cell cycle, DNA replication, the spliceosome, repair, and metabolism-related processes were all significantly enriched in the high-risk group. Among the enriched genes, the expression levels of the replication protein RPA1 and the pre-mRNA splicing factor SF3B1 were significantly upregulated in the high-risk group. These results might help in elucidating the underlying molecular mechanisms of HBV-related HCC.

**Conclusions:**

Our data may provide new predictive signatures and potential therapeutic targets to identify and treat HBV-related HCC patients in the early disease stage.

## Background

N6-methyladenosine (m6A), which occurs in the messenger RNAs (mRNAs) of most eukaryotes, is the most prevalent modification of mammalian RNA (Desrosiers et al. 1974). This modification is mediated by a series of protein factors, including the writer complex subunits (*METTL3, METTL14, WTAP, RBM15, KIAA1429*, and *ZC3H13*), erasers (FTO and ALKBH5), and readers (YTHDF1/2/3, YTHDC1/2, eIF3, IGF2BP1/2/3, HNRNPA2B1, FMR1, and LRPPRC) (Wang et al. 2018a). These m6A regulators exert enormous influence on cancer development processes such as proliferation, migration, and invasion (Liu et al. 2018). In glioblastoma, WTAP was identified as an independent prognostic factor; moreover, high expression of WTAP predicts poor overall patient survival (Xi et al. 2016). Another study found that YTHDC2 acts as a promoter in colon cancer metastasis and may be a diagnostic marker for colon cancer patients (Tanabe et al. 2016).

Hepatocellular carcinoma (HCC) has the sixth-highest incidence of malignant tumors and is the fourth most deadly cancer worldwide (Torre et al. 2015). Hepatitis B viral (HBV) infection is still the leading cause of liver cirrhosis and hepatocellular carcinomas (Villanueva 2019). While HBV vaccination has reduced the incidence of hepatocellular carcinoma, there are still many people who have not been vaccinated against HBV, especially in Asia, and who are thus at a higher risk of HBV infection (Chang et al. 2009). Active hepatitis B continues to account for the majority of the global HCC burden (Kulik and El-Serag 2019). It was suggested that the occurrence and development of HCC is a multistep process involving complex interactions between genetic, epigenetic, and transcriptional changes (Wong et al. 2003). Several studies have pointed out that hepatocarcinogenesis is correlated with abnormal m6A modifications (Chen et al. 2018; Ma et al. 2017). Chen et al. found that high METTL3 expression in human HCC leads to high m6A levels in SOCS2 mRNA, which causes the rapid degradation of SOCS2 and eventually leads to HCC occurrence (Chen et al. 2018). Ma et al. found that METTL14 had no remarkable effect on HCC; however, METTL14 downregulation was correlated with poor prognosis in HCC patients without recurrence (Ma et al. 2017).

Hepatitis B infection is the main cause of HCC, but it is unknown whether m6A regulators could be potential biomarkers for prognosis in HBV-related HCC. Here, we mined the expression pattern of m6A regulators in HBV-related HCC samples using the TCGA database. We conducted Lasso Cox regression analysis and identified the two-gene signature of *RBM15* and *HNRNPA2B1* as a novel prognostic biomarker. The time-dependent receiver operating characteristic (ROC), along with univariate and multivariate Cox regression analysis, confirmed the independent prognostic role of this two-gene signature. In addition, the prognostic value of *RBM15* and *HNRNPA2B1* was further validated in the GSE14520 dataset. Survival analysis of the Guilin cohort indicated that the two-gene signature might be a predictor for patients in the early TNM stage; stratification analysis based on the TNM stage in the TCGA and GSE14520 cohorts also validated this result.

## Methods

### Gene datasets and clinical data collection

Level 3 mRNA expression and clinical data of samples, including 374 liver hepatocellular carcinoma (LIHC) and 50 healthy control samples, were obtained from the TCGA database (https://portal.gdc.cancer.gov/). The expression level of all probes was first normalized using the fragments per kilobase of exon per million reads mapped (FPKM) method, and then underwent a log2 transformation. After studying the clinical information to determine the viral histories of the patients, 93 HBV-related HCC (containing both survival time and survival state) and 50 healthy samples were selected for further analysis. The GSE14520 dataset from the gene expression omnibus (GEO) database, which contains 224 HBV-related HCC cases, was downloaded to validate our results from TCGA further. Both TCGA and GEO data were obtained from public databases; thus, there was no need for additional ethics approval.

We collected 22 paired tumor and para-tumor HCC cases who underwent hepatectomy at the Affiliated Hospital of Guilin Medical University (Guilin, People’s Republic of China) between May 2002 and September 2010. The Guilin dataset patients were diagnosed according to clinical information, serological testing, abdominal ultrasound examinations, and magnetic resonance imaging. Diagnoses were verified using pathologic examination. Eighty-five percent (19/22) of patients were followed up from diagnosis until death. The sample collection methodology was confirmed by the Research Ethics Committee of the Affiliated Hospital of Guilin Medical University. Written informed consent was obtained from all patients before sample collection at the Affiliated Hospital of Guilin Medical University.

The clinical characteristics of the TCGA, GSE14520, and Guilin datasets are summarized in Table 1. After studying the available literature regarding the advances in mRNA m6A, we selected 21 m6A regulators for further model building, including *METTL3, METTL14, METTL16, WTAP, KIAA1429, RBM15, ZC3H13, YTHDC1, YTHDC2, YTHDF1, YTHDF2, YTHDF3, HNRNPC, HNRNPA2B1, FTO, ALKBH5, IGF2BP1, IGF2BP2, IGF2BP3, FMR1*, and *LRPPRC* (Wang et al. 2018a).

**Table 1:** Clinical characteristics of TCGA, GSE14520 and Guilin cohorts

	TCGA (93)	GSE14520 (224)	Guilin cohort (22)
Survival state			
Dead	18 (19.4%)	86 (38.4%)	19 (86.4%)
Alive	75 (80.6%)	138 (61.6%)	3 (13.6%)
Age			
< 65	70 (75.3%)	199 (88.8%)	20 (90.9%)
> = 65	23 (24.7%)	25 (11.2%)	2 (9.1%)
Gender			
Female	15 (16.1%)	29 (12.9%)	0
Male	78 (83.9%)	195 (87.1%)	22 (100%)
TNM stage			
I + II	84 (90.3%)	174 (77.7%)	4 (18.2%)
III + IV	9 (9.7%)	50 (22.3%)	18 (81.8%)
Metastasis Signature			
YES	none	108 (48.2%)	11 (50%)
NO		116 (51.8%)	11 (50%)

### Differential analysis

The m6A regulators and patient survival information from the clinical data were processed, extracted, and integrated using custom Perl scripts (https://www.perl.org/). The m6A regulators differentially expressed between tumor tissues and healthy tissues were calculated and labeled using the “Limma” package.

### Establishment of the prognostic gene signature

Next, univariate Cox regression analysis was performed to confirm prognostic genes; genes were considered significant at *P* < 0.05. The least absolute shrinkage and selection operator (Lasso) Cox regression analysis was performed to select prognostic genes for overall survival (OS) analysis in the HBV-related HCC dataset using the R packages “survival” and “glmnet.” Finally, we selected two genes to establish a prognostic risk signature. The optimal cutoff value was identified using the R package “survminer” and a two-sided log-rank test. Using the optimal risk score as the threshold, the patients were separated into high-risk and low-risk groups according to the risk score, which was calculated with the equation:

$$ {\displaystyle \begin{array}{l}\mathrm{Risk}\ \mathrm{score}=\Big(\mathrm{coefficient}\ \mathrm{mRNA}1\ast \mathrm{expression}\ \mathrm{level}\ \mathrm{of}\\ {}\mathrm{mRNA}1\left)+\right(\mathrm{coefficient}\ \mathrm{mRNA}2\ast \mathrm{expression}\ \mathrm{level}\ \mathrm{of}\\ {}\mathrm{mRNA}2\left)+\right(\mathrm{coefficient}\ \mathrm{mRNA}3\ast \mathrm{expression}\ \mathrm{level}\ \mathrm{of}\\ {}\mathrm{mRNA}3\Big)+\cdots +\left(\mathrm{coefficient}\ \mathrm{mRNA}\mathrm{n}\ast \mathrm{expression}\ \mathrm{level}\ \mathrm{of}\ \mathrm{mRNA}\mathrm{n}\right)\end{array}} $$

The Kaplan–Meier survival and ROC curves were drawn to evaluate the predictive value of the prognostic gene signature for OS using the R packages “survival” and “survivalROC,” respectively. We also validated whether risk group analysis was significantly correlated with clinical information. Moreover, correlation analysis between the two genes was executed using the R package “corrplot.”

### Identification of the independent prognostic role of the gene signature

We further explored the independent prognostic role of the prognostic gene signature with univariate and multivariate analyses using the Cox regression model method with the “survival” R package. We also incorporated other factors such as gender, age, tumor grade, alcohol consumption, and cancer stage, in this step. A *P* value < 0.05 was considered statistically significant.

### Gene Set Enrichment Analyses (GSEA)

Here, patients in the TCGA dataset were divided into two groups (high-risk and low-risk) according to the established m6A modification-related gene model. We performed GSEA to explore the underlying mechanisms using all 20,530 genes. The Kyoto Encyclopedia of Genes and Genomes (KEGG) gene set “c2.cp.kegg.v7.0.symbols.gmt” was used for further analysis. A *P* value of < 0.01 and FDR (false discovery rate) q value of < 0.25 were considered statistically significant.

### External validation of the genes in the prognostic gene signature

The GSE14520 dataset from the GEO database was used to validate the gene signature. A Kaplan–Meier curve was constructed to test the predictive value of the gene signature. The relationships between mRNA expression and age, gender, AFP, TNM stage, and metastasis risk were analyzed. Images were produced, and statistical analysis was performed using GraphPad Prism 5.0 and R software.

### Quantitative real-time PCR

Relative quantitation of the 22 paired mRNAs was performed using quantitative real-time polymerase chain reaction (qRT-PCR) analysis (LightCycler 480 SYBR Green I Master, Thermo Fisher) to determine whether the two genes had a predictive role in our Guilin dataset (HBV-related HCC patients with tumor and para-tumor tissues). Amplification reactions were performed in a volume of 20 μl under the following conditions: 95 °C for 5 min, followed by 45 cycles of 95 °C for 30 s, 55 °C for 20 s, and 77 °C for 30 s, followed by 42 °C for 10 s. *RBM15*-specific primers were as follows: forward primer, (5′- ACCGCAGTCCAGAATTGAGC-3′); reverse primer, (5′- ACTTCAGCTTGGAGGAAGCAG-3′) (position: 1948–2206, length: 279 bp). HNRNPA2B1-specific primers were used as follows: forward primer, (5′- GCAGGAAGTTCAGAGTTCTAGG-3′); reverse primer, (5′- AGTTACTTCCTGGTCCTGGTC-3′) (position: 748–828, length: 101 bp). GAPDH-specific primers were used as follows: forward primer, (5′- GTCTTCACCACCATGGAGAAG-3′); reverse primer, (5′- CATGAGTCCTTCCACGATACC-3′) (position: 323–527, length: 225 bp). Data analysis was calculated using the ΔΔCT method.

### Statistical analysis

Statistical analyses were performed using R software v3.6.0. Qualitative variables were analyzed using the Pearson χ2 test. A *P* value < 0.05 indicated statistical significance.

## Results

### Identification of the differentially expressed m6A regulators

**Fig. 1:**
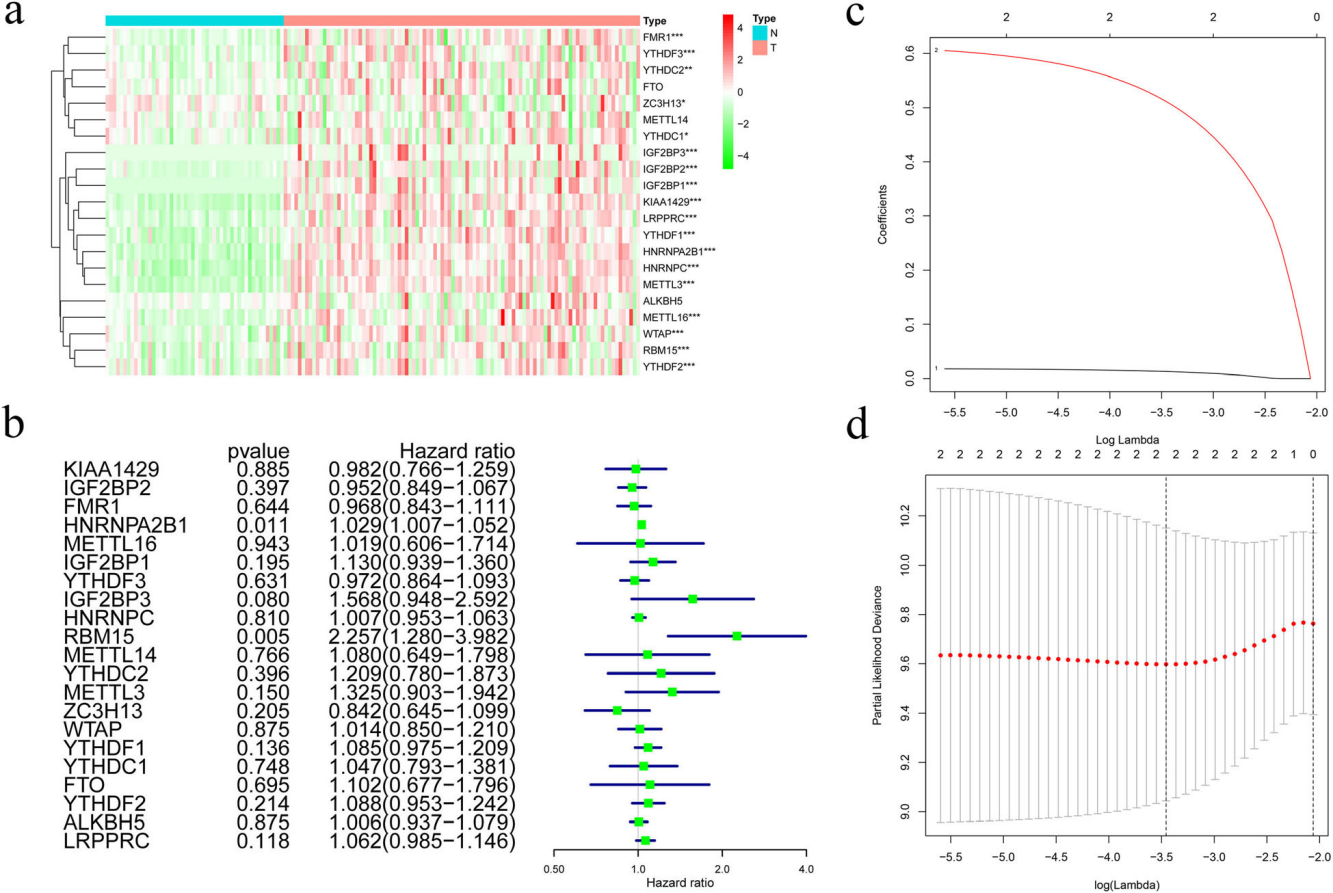
**a** Heatmap of m6A-related gene expression in 374 HBV-related tumor tissues and 50 normal tissues. “*” represents statistical significance at *P* < 0.05, “**” represents statistical significance at *P* < 0.01, and “***” represents statistical significance at *P* < 0.001. **b** Forest plot of the univariate Cox regression analysis in m6A-related genes. **c** and **d** Establishment of the Lasso regression model

Out of the 21 m6A regulatory genes, 18 differentially expressed genes were identified in the tumor tissues (*n* = 93) and healthy tissues (*n* = 50) in the TCGA dataset. *P* < 0.05 indicated statistical significance. The heatmap and the differentially expressed m6A regulators are shown in Fig. 1a and Table S1.

### Establishment and validation of the two-gene-based prognostic gene signature

Two genes (*HNRNPA2B1* and *RBM15*) were significantly correlated with OS (*P* < 0.05) by using Cox univariate analysis (Fig. 1b and Table S2). These two genes were identified using Lasso Cox and subsequently used to build a prognostic gene signature in the TCGA dataset, which was used as a training set (Fig. 1c and d; Table S3). We calculated the two-gene based risk score for each patient using the equation:

Risk score = 0.0125 * Expression *HNRNPA2B1*+ 0.5117 * Expression *RBM15*.

**Fig. 2:**
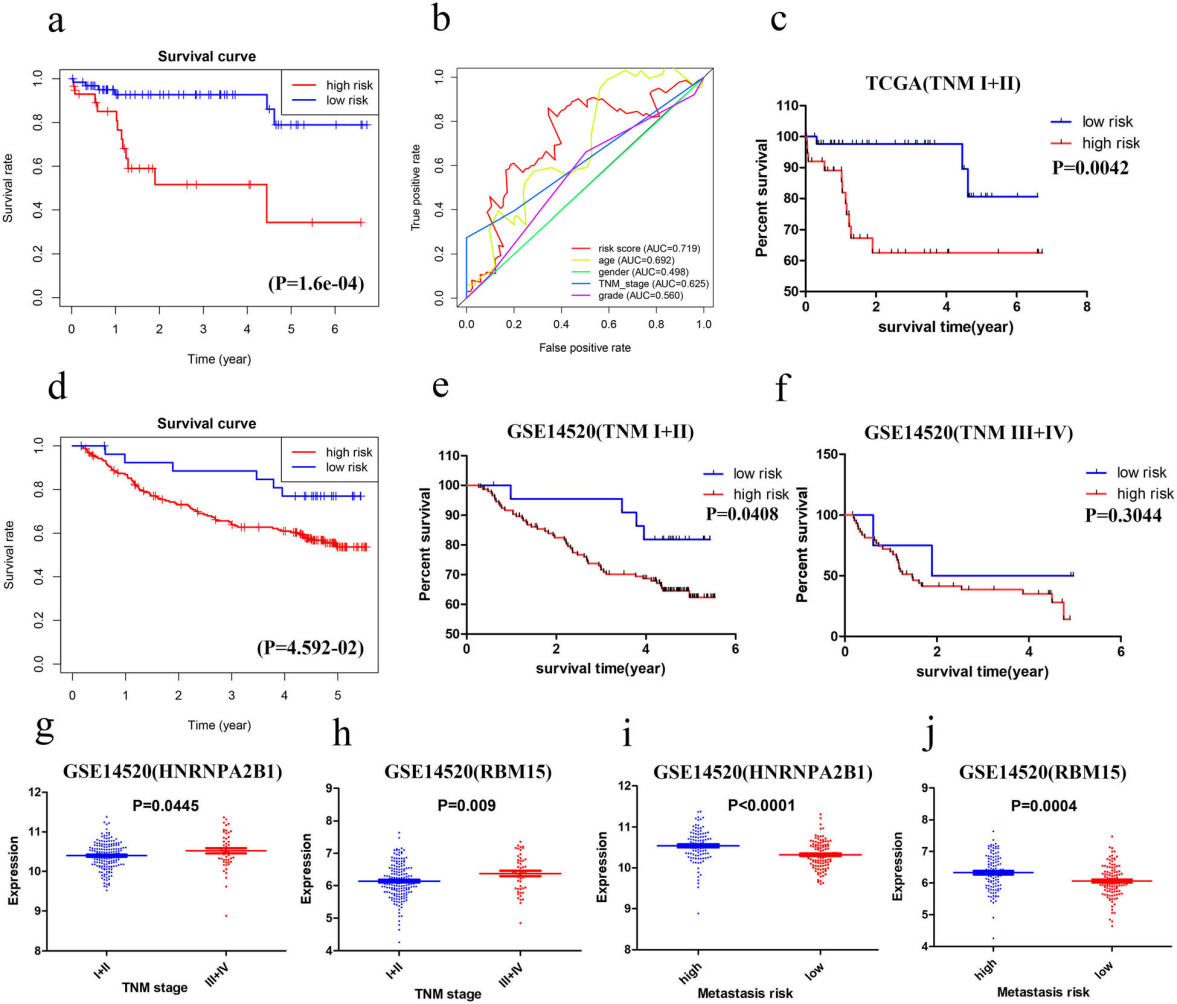
Kaplan–Meier analysis of the high-risk and low-risk groups in HBV-related HCC in the TCGA cohort (**a**). The ROC curves of the risk score, age, gender, TNM stage and grade in HBV-related HCC (**b**). Kaplan–Meier analysis of the two groups (high-risk and low-risk) for patients with HBV-related HCC at early TNM stages (**c**). In the GSE14520 cohort, Kaplan–Meier analysis of the two groups (high-risk and low-risk) for patients with HBV-related HCC (**d**) and for patients at early TNM stages (**e**) and late TNM stages (**f**). Expression of HNRNPA2B1 and RBM1 based on the GSE14520 dataset (**g-j**)

We subsequently divided all the patients in the TCGA dataset into the high-risk or low-risk group based on the optimal cutoff point of the risk score. The Kaplan–Meier survival and ROC curves were drawn to evaluate the prognostic capacity of the two-gene signature. Patients in the high-risk group had significantly worse OS than those in the low-risk group (*P* < 0.0001) (Fig. 2a). The AUCs (Area under the ROC curve) for the risk score, TNM stage, gender, age, and grade were 0.719, 0.625, 0.498, 0.692, and 0.560, respectively, as shown in Fig. 2b and Table S4.

The GSE14520 dataset (including 221 HBV-related HCC patients) from the GEO was used to verify the predictive value of the two-gene signature. The same coefficients were used to calculate the risk score for the GSE14520 dataset. Based on the optimal cutoff value, we also divided the patients into a high-risk group, and a low-risk group, patients in the high-risk group had poorer overall survival than those in the low-risk group (*P* = 0.04592; Fig. 2d).

**Fig. 3:**
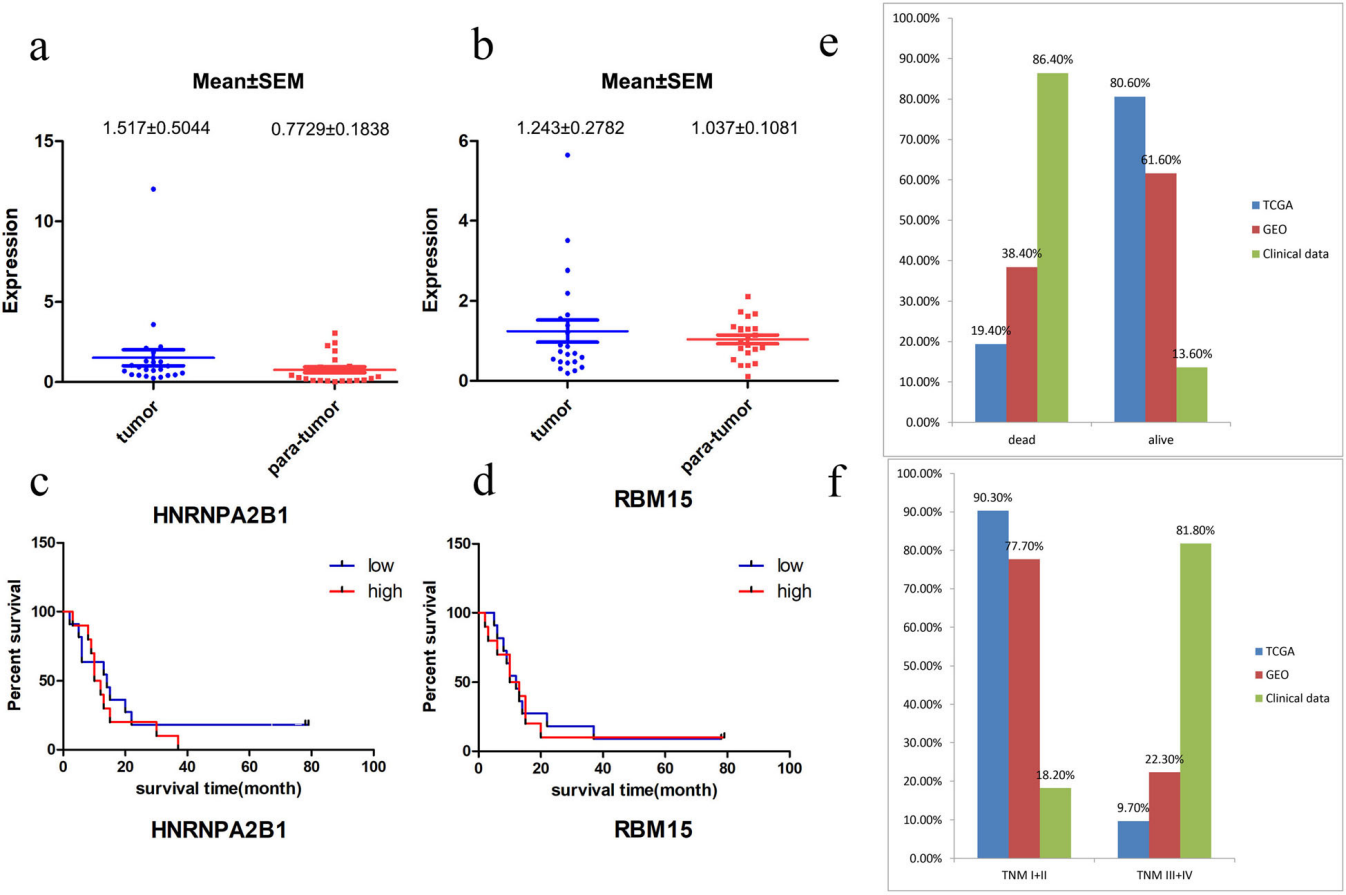
HNRNPA2B1 and RBM15 mRNA expression measured by qRT-PCR; corresponding survival curves in the Guilin cohort. **a** The mRNA expression of HNRNPA2B1 in the tumor and para-tumor groups. **b** The mRNA expression of RBM15 in the tumor and para-tumor groups. **c** Survival curve of HNRNPA2B1 in the Guilin cohort. **d** Survival curve of RBM15 in the Guilin cohort. **e** Case distribution related to TNM stage (I + II vs III + IV) in the TCGA, GSE14520, and Guilin cohorts. **f** Case distribution related to survival state (dead vs. alive) in the TCGA, GSE14520, and Guilin cohorts

Twenty-two patients with HBV-related HCC were collected retrospectively to form our Guilin dataset. We used the qRT-PCR method to measure both the *HNRNPA2B1* and *RBM15* mRNA expression in tumorous and para-tumorous tissues. As shown in Fig. 3a and b, the mRNA expression of the two genes was higher in tumorous tissue than in para-tumorous tissue (with no significant *P*-value). Survival curves for the Guilin dataset showed that the *P-*value for the difference in OS was not significant (Fig. 3c and d), which could be attributed to the differences in clinical information among the three datasets (shown in Table 1 and Fig. 3e and f). The analysis showed that 86.4% of cases in the Guilin dataset were followed up to death, whereas only 19.4% in the TCGA dataset and 38.4% in the GSE14520 dataset were followed up to death. Moreover, 81.1% of patients in the Guilin dataset were in the late TNM stage (TNM III + IV stage), whereas only 9.7% in the TCGA dataset and 22.3% in the GSE14520 dataset were in the late stage. More samples and further investigations are needed to validate the predictive role of the identified gene signature.

Based on the results of the Guilin dataset, we performed a stratified TNM stage analysis. In the TCGA dataset, for patients in the early TNM stage (I + II), the survival of the low-risk group was significantly higher than that of the high-risk group (*P* = 0.0042; Fig. 2c). The late TNM stage (III + IV) was not analyzed because of the small sample size (ten cases in total). In the GSE14520 dataset, for patients in the early TNM stage, the survival of the low-risk group was significantly higher than that of the high-risk group (*P* = 0.0408) (Fig. 2e). There was no significant difference in the OS between the two groups for patients in the late TNM stage (Fig. 2f). Furthermore, the expression of both *HNRNPA2BA* and *RBM15* had a close relationship with the TNM stage and metastasis risk. For *HNRNPA2B1*, patients in the TNM III + IV stage and the high metastasis risk group had a higher mRNA expression than patients in TNM I + II stage and in the low metastasis risk group, with *P* values of 0.0445 and < 0.0001, respectively (Fig. 2g and h). *RBM15* expression showed a similar trend, with *P* values of 0.009 and 0.0004, respectively (Fig. 2i and j). In general, our results showed that the two-gene signature acted as a prognostic indicator for HBV-related HCC patients in the early TNM stage.

### Independent prognostic role of the gene signature

**Fig. 4:**
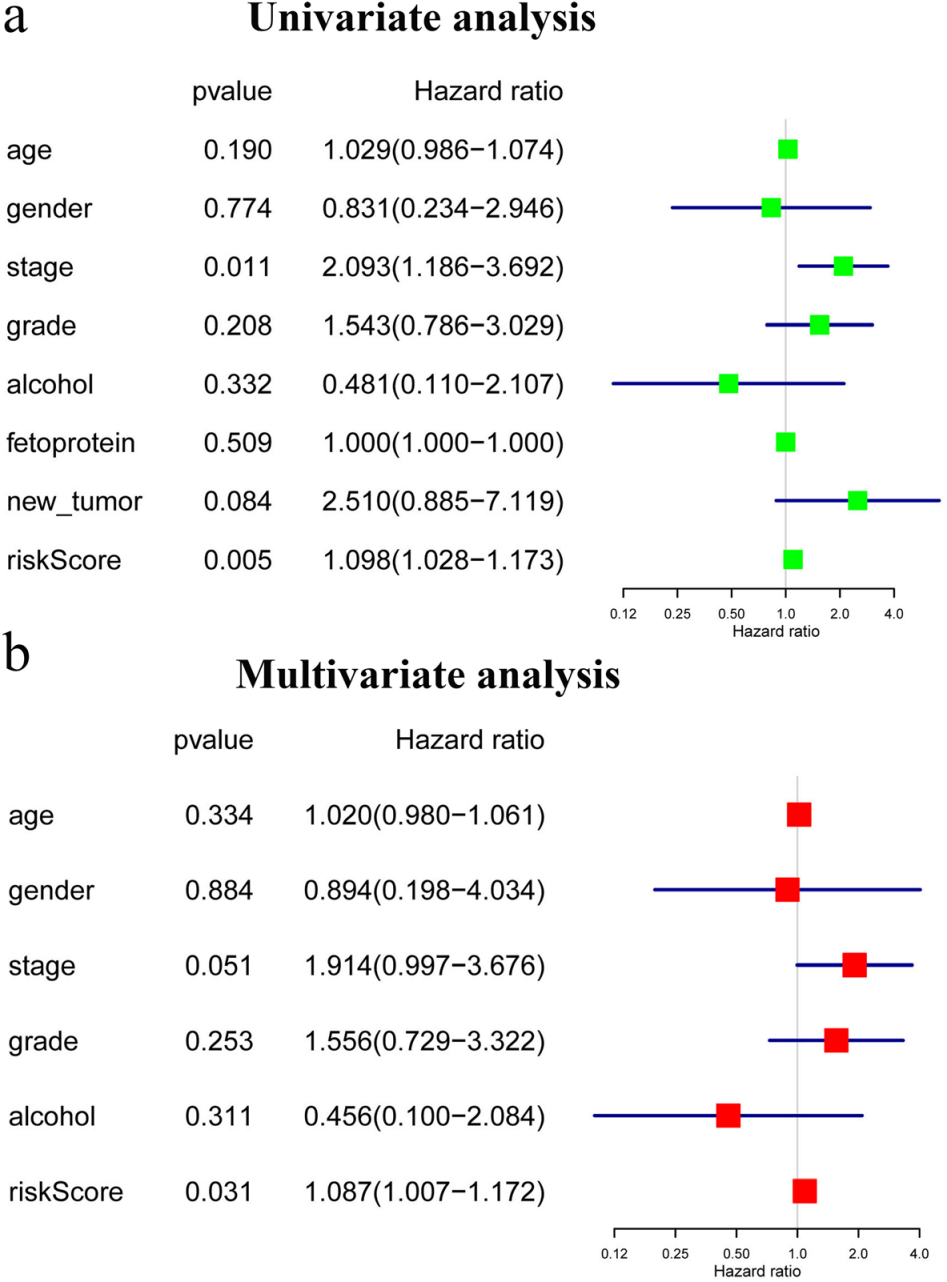
Forest plot of the univariate and multivariate Cox regression analysis in HBV-related HCC. **a** Univariate regression analysis in HBV-related HCC. **b** Multivariate Cox regression analysis in HBV-related HCC

We performed univariate and multivariate Cox regression analysis to confirm whether the two-gene signature could be an independent prognostic factor for patients with HBV-related HCC. Ninety-three HBV-related HCC patients in the TCGA dataset with complete information, including gender, age, AFP, stage, tumor grade, alcohol consumption, and new tumor events, were included for further analysis (Table 2). Univariate analysis indicated that the TNM stage and the risk score were significantly associated with OS, with *P-*values of 0.011 and 0.005, respectively (Fig. 4a). Multivariate Cox regression analysis showed that the risk score calculated from the two-gene signature was an independent predictor for OS (*P-*value =0.031; Fig. 4b and Table S5 and S6).

**Table 2:** Correlation of clinic pathologic characteristics and the two-gene signature in HBV-related HCC in TCGA cohort

	HBV-related HCC (*n* = 93)		
Characteristics	High risk	Low risk	*P*.Value
Gender			0.77
Male	37	6	
Female	9	41	
Age (years)			0.19
< =65	35	38	
> 65	11	10	
TNM stage			0.01
I + II	40	44	
III + IV	6	3	
Tumor grade			0.21
1 + 2	12	29	
3 + 4	34	22	
Alcohol assumption			0.33
YES	8	15	
NO	38	32	
AFP			0.51
< 300	37	33	
> =300	9	14	
New_tumor			0.08
YES	27	12	
NO	19	35

### Correlation analysis

**Fig. 5:**
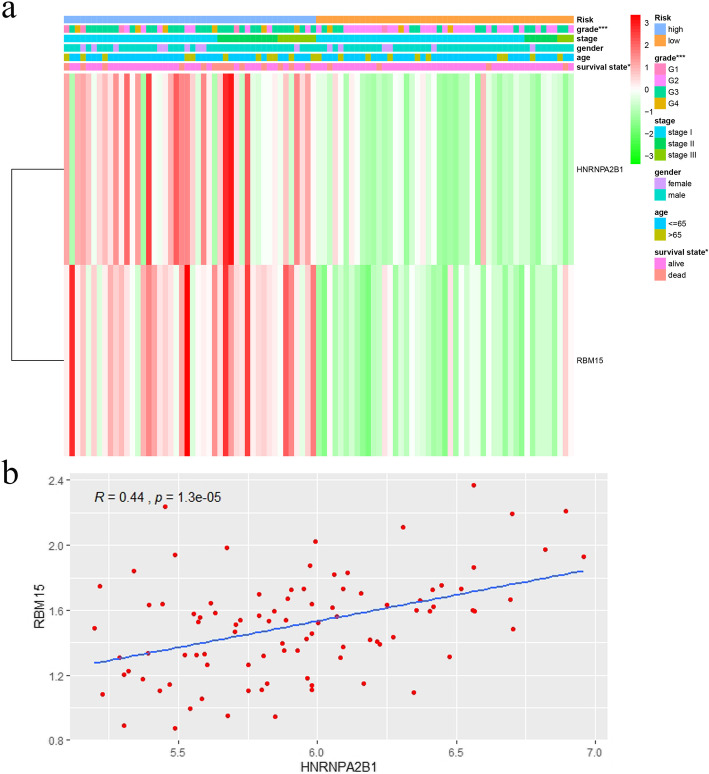
**a** Heatmap of RBM15 and HNRNPA2B1 expression; correlation between expression and clinical information in the high-risk and low-risk groups. “*” represents statistical significance at *P* < 0.05, “**” represents statistical significance at *P* < 0.01, “***” represents statistical significance at *P* < 0.001. **b** Correlation analysis between RBM15 and HNRNPA2B

As shown in Fig. 5a, the risk group analysis was significantly correlated with grade and survival state in the TCGA dataset. This finding indicated that with grade 1–2, the survival state (alive), and low expression of the two genes (*HNRNPA2B1* and *RBM15*) were more common in the low-risk group. Grade 3–4, the survival state (dead), and high expression of the two genes were significantly related to the high-risk group. In addition, correlation analysis indicated that *HNRNPA2B1* and *RBM15* expression was significantly correlated (correlation coefficient = 0.44, *P* < 0.0001) (Fig. 5b).

### Gene set enrichment analyses

**Fig. 6:**
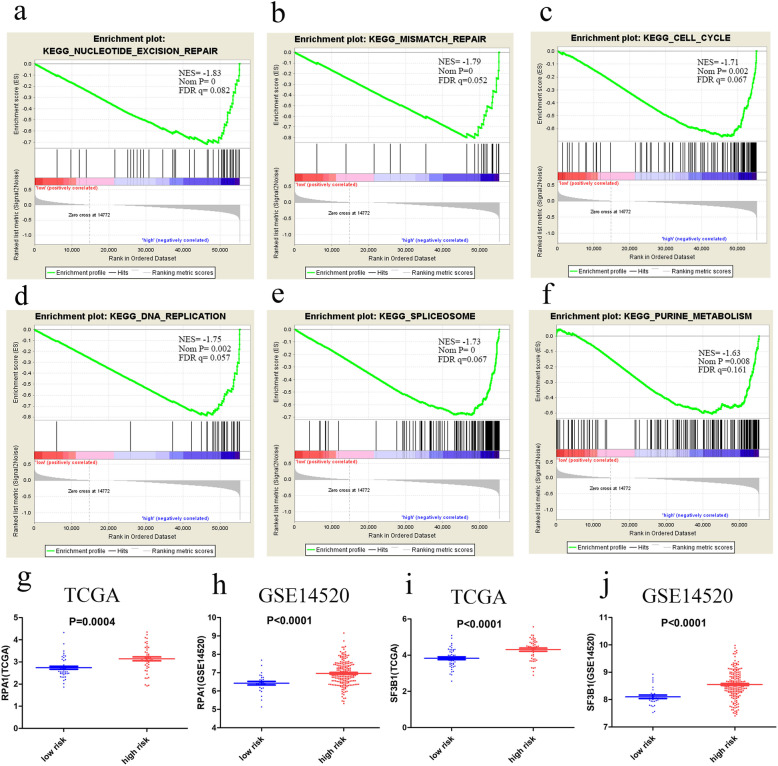
KEGG pathways identified in the high-risk group; *P* < 0.05 and FDR q < 0.25 were considered significant (**a-f**). The expression of enriched genes in the different risk groups, based on the TCGA and GSE14520 datasets (**g-j**). (FDR, false discovery rate)

KEGG pathway analysis in the high-risk group suggested that some critical biological processes, including the cell cycle, DNA replication, spliceosome, repair mechanisms (mismatch repair, nucleotide excision repair), and pyrimidine metabolism, were enriched (Fig. 6a-f). We subsequently analyzed the expression of the enriched genes from the above KEGG pathways in the different risk groups in the TCGA and GSE14520 datasets. The analyzed genes are listed in Table S7. Among these genes, *RPA1* was enriched in three pathways (nucleotide excision repair, mismatch repair, and DNA replication), ranking first or second in terms of differential expression. The results showed that *RAP1* and *SF3B1* mRNA expression was significantly upregulated in the high-risk group in both the TCGA (*P* ≤ 0.0004 and < 0.0001, respectively) and the GSE14520 datasets (*P* < 0.0001) (Fig. 6g-j).

## Discussion

The incidence and mortality of HCC increase every year, owing to a lack of precise diagnosis in the early-stages (Kudo 2013; Ichikawa et al. 2014). Conventional methods, such as TNM staging, grading, vascular invasion, and AFP value cannot meet the demands required for early HCC prognosis prediction. With the rapid development of high-throughput sequencing, more genes and their functional mechanisms have been identified and used as predictive biomarkers. For hepatocellular carcinoma, Yan et al. identified a four-gene-based signature as an OS predictor in HCC patients (Yan et al. 2019). Ma et al. constructed a prognostic four lncRNA model in patients with cirrhotic HCC (Ma and Deng 2019). Liu et al. identified a four-gene metabolic signature for HCC prognosis prediction (Liu et al. 2019). Long et al. confirmed four gene biomarkers to predict HCC patient survival (Long et al. 2018). Shen et al. also indicated that SF3B4 functions as an early-stage driver in liver cancer development (Shen and Nam 2018). All these studies focused on all types of HCC. Many types of chronic liver disease, including hepatitis B or C viral (HBV or HCV) infection, alcohol abuse, and nonalcoholic fatty liver disease (NAFLD) can lead to HCC. This difference in pathogenesis can lead to differences in their underlying mechanisms (Villanueva 2019). Nevertheless, HBV remains the leading cause of liver cirrhosis and hepatocellular carcinomas. Therefore, it is essential to explore potential biomarkers in HBV-related HCC patients (Suk-Fong 2019).

Previous studies have indicated that m6A modification plays a role in HCC occurrence and development (Liu et al. 2018; He et al. 2019; Dai et al. 2018). Here, we aimed to identify an m6A RNA methylation regulator signature as a prognostic molecular biomarker for HBV-related HCC patients. It is worth mentioning that the two identified genes (*HNRNPA2B1* and *RBM15*) did not show a statistically significant correlation with survival in the Guilin dataset, but did in the other two datasets. After we summarized the clinical characteristics in the three datasets, we found that both the TCGA and GSE14520 datasets had a similar distribution of clinical information characteristics. Our Guilin dataset had a distribution opposite the TNM stage and survival state characteristics. In other words, the TCGA and GSE14520 datasets agreed in terms of clinical characteristics, but they both differed from the Guilin dataset. These factors might influence the consistency of the outcome, or the gene signature possibly might play a vital role in the prognosis of HBV-related HCC patients in the early stage. We subsequently performed a hierarchical analysis of the TNM stage in the TCGA and GSE14520 datasets. These results showed that the gene signature should act as a useful prognostic indicator for HBV-related HCC patients in the early TNM stage. Meanwhile, the signature was found to be a negative prognostic marker in patients with HBV-related HCC in our three datasets. Furthermore, risk group analysis based on the gene signature was also significantly correlated with the grade and survival state in the TCGA dataset, which supported the established gene signature for estimating the OS of HBV-related HCC. A previous study found that *HNRNPA2B1* promoted the NF-κB signaling pathway to facilitate tumor metastasis in liver cancer (Wang et al. 2018b).

GSEA analysis for the high-risk group showed that the selected genes are associated with the cell cycle, DNA replication, the spliceosome, DNA repair, and pyrimidine metabolism; the result has a close relationship with the role of m6A modification (Liu and Zhang 2018; Wu et al. 2018). By comparing the expression of the related genes in the different risk groups, the expression of the replication protein RPA1 and the pre-mRNA splicing factor SF3B1 was found to be upregulated in the high-risk group. A recent study identified the anti-SPF3B1 autoantibody as a diagnostic biomarker in patients with HCC using an HCC model HBx-transgenic mouse. This finding indicated that SF3B1 might be a tumor-associated antigen that is targeted to activate the immune system (Hwang et al. 2018). RPA1 is one of the members that constitute the canonical RPA heterotrimer, which is crucial for genome maintenance and cell proliferation (Fanning et al. 2006; Ishibashi et al. 2006). Previous studies have shown that RPA1 is upregulated in colon cancer and esophageal carcinoma; moreover, it is related to the extent of illness (Givalos et al. 2007; Dahai et al. 2013). Another study reported that RPA1 was increased in both liver cancer cell lines and HCC tissues and promoted the proliferation of HCC via the cyclin-dependent-kinase 4(CDK4)/cyclin-D pathway (Wang et al. 2018c). More research is needed to elucidate the potential mechanisms by which RPA1 and SF3B1 may influence outcomes in patients with HBV-related HCC.

M6A RNA methylation is the most abundant mRNA modification in mammalian cells and plays a vital role in tumor development and progression (Roundtree et al. 2017). The development of high-throughput sequencing technology paves the way for the study of mRNA modification, but different high-throughput detection techniques are error-prone to varying degrees (Helm and Motorin 2017). A recent study implied that to date, the published evidence remains insufficient to support the presence of DNA N6-methyladenine (6 mA) in mammals because of erroneous detection, including bacterial and RNA contamination, technological limitations, and antibody non-specificity (Douvlataniotis et al. 2020). Although the relationship between DNA 6 mA and RNA m6A remains unclear, it is essential to validate the m6A modified sites predicted from big data with at least one additional independent method (Helm and Motorin 2017). In our study, we aimed to identify the m6A RNA methylation regulator gene signature to provide predictive value for HBV-related HCC patients. However, there were several limitations to this study. One limitation is that we did not directly measure whether m6A modifications were associated with HCC prognosis. Since it was not the aim of our research, we only assessed whether the expression of regulatory genes related to m6A modifications was associated. Therefore, based on this study, it is not possible to confirm that m6A RNA methylation itself is related to HCC prognosis. The second limitation is the small sample size of the Guilin cohort, and more cases are required to validate our results. A third limitation is that we did not identify the genes which are required for the metabolic cascade to produce the m6A modification. Further study is required to develop and validate the prognostic model based on the genes that indirectly affect m6A RNA methylation.

## Conclusions

Based upon the accuracy of the established model for estimating OS in patients with HBV-related HCC in the early TNM stage, our study paves the way for further investigations on both *RBM15* and *HNRNPA2B1* as prognostic biomarkers and therapeutic targets. Nevertheless, more independent HBV-related HCC cohorts are necessary to validate the predictive role of the established model. Further studies are essential to clarify the specific impact of *RBM15* and *HNRNPA2B1* in tumorigenesis.

## Supplementary information

**Additional file 1: Table S1.** Differentially expressed m6A RNA methylation regulators were identified in tumor tissues when compared with normal tissues.

**Additional file 2: Table S2.** Univariate Cox regression analysis was constructed to confirm prognostic genes, and genes were considered significantly with a cut-off of P. Value < 0.05.

**Additional file 3: Table S3.** The coefficient of the identified genes.

**Additional file 4: Table S4.** Comparison of the riskScore model with stage, gender, age, and grade models.

**Additional file 5: Table S5.** Identification of the independent prognostic role of prognostic gene signature by univariate analyses using the Cox regression model.

**Additional file 6: Table S6.** Identification of the independent prognostic role of prognostic gene signature by multivariate analyses using the Cox regression model.

**Additional file 7: Table S7.** The enriched genes in KEGG pathways.

## Data Availability

All data not included in the manuscript is available from the supplementary materials.

## Abbreviations

m6A: N6-methyladenosine
HCC: Hepatocellular carcinoma
HBV: Hepatitis B virus
TCGA: The Cancer Genome Atlas
GEO: Gene expression omnibus
KEGG: Kyoto Encyclopedia of Genes and Genomes
GSEA: Gene Set Enrichment Analyses
