# Supplementary material for: The significance of m6A RNA methylation regulators in predicting the prognosis and clinical course of HBV-related hepatocellular carcinoma
Source: Mol Med. 2020 Jun 17;26:60. doi: 10.1186/s10020-020-00185-z (PMC7302147; doi:10.1186/s10020-020-00185-z)
Supplement: Supplementary file 1 — Additional file 1: Table S1. Differentially expressed m6A RNA methylation regulators were identified in tumor tissues when compared with normal tissues. [file 10020_2020_185_MOESM1_ESM.docx]

| Table S1. Differentially expressed m6A modification related genes were identified in tumor tissues when compared with normal tissues. | | | | |
| --- | --- | --- | --- | --- |
| gene | conMean | treatMean | logFC | pValue |
| KIAA1429 | 2.038047 | 4.831909 | 1.245406 | 2.31E-18 |
| IGF2BP2 | 0.520433 | 4.176433 | 3.004486 | 6.11E-05 |
| FMR1 | 3.610126 | 6.218541 | 0.784527 | 6.31E-07 |
| HNRNPA2B1 | 35.39061 | 63.54614 | 0.844438 | 1.05E-19 |
| METTL16 | 0.958741 | 1.520078 | 0.664932 | 6.66E-08 |
| IGF2BP1 | 0.009142 | 1.794541 | 7.616863 | 1.54E-14 |
| YTHDF3 | 7.164098 | 10.07169 | 0.491449 | 7.50E-06 |
| IGF2BP3 | 0.010405 | 0.421368 | 5.339767 | 6.77E-09 |
| HNRNPC | 19.33566 | 34.28524 | 0.826324 | 1.87E-18 |
| RBM15 | 1.472535 | 1.959026 | 0.411834 | 9.90E-06 |
| YTHDC2 | 1.568341 | 2.051241 | 0.387258 | 0.001715 |
| METTL3 | 1.366178 | 3.302807 | 1.273547 | 3.70E-17 |
| ZC3H13 | 3.47123 | 3.348379 | -0.05198 | 0.039863 |
| WTAP | 5.134045 | 6.814891 | 0.408595 | 0.000102 |
| YTHDF1 | 9.060679 | 14.49496 | 0.67786 | 4.51E-15 |
| YTHDC1 | 4.251735 | 4.931946 | 0.214105 | 0.025446 |
| YTHDF2 | 8.640147 | 11.03449 | 0.352892 | 3.07E-05 |
| ALKBH5 | 18.68256 | 23.18662 | 0.3116 | 0.084661 |
| LRPPRC | 7.690573 | 12.82319 | 0.737592 | 2.89E-11 |
