# Supplementary material for: The significance of m6A RNA methylation regulators in predicting the prognosis and clinical course of HBV-related hepatocellular carcinoma
Source: Mol Med. 2020 Jun 17;26:60. doi: 10.1186/s10020-020-00185-z (PMC7302147; doi:10.1186/s10020-020-00185-z)
Supplement: Supplementary file 2 — Additional file 2: Table S2. Univariate Cox regression analysis was constructed to confirm prognostic genes, and genes were considered significantly with a cut-off of P. Value < 0.05. [file 10020_2020_185_MOESM2_ESM.docx]

| Table S2. Univariate Cox regression analysis was constructed to confirm prognostic genes, and genes were considered significantly with a cut-off of P.Value < 0.05. | | | | |
| --- | --- | --- | --- | --- |
| id | HR | HR.95L | HR.95H | pvalue |
| KIAA1429 | 0.981879 | 0.765891 | 1.2587775 | 0.8852824 |
| IGF2BP2 | 0.9519345 | 0.8493412 | 1.0669201 | 0.3971992 |
| FMR1 | 0.967968 | 0.8430623 | 1.1113794 | 0.6441853 |
| HNRNPA2B1 | 1.0290596 | 1.0066672 | 1.0519501 | 0.0107117 |
| METTL16 | 1.019219 | 0.6061861 | 1.7136773 | 0.9427555 |
| IGF2BP1 | 1.1300215 | 0.9391829 | 1.3596379 | 0.1952676 |
| YTHDF3 | 0.9716241 | 0.8640427 | 1.0926005 | 0.6306612 |
| IGF2BP3 | 1.5675176 | 0.947907 | 2.5921437 | 0.0798598 |
| HNRNPC | 1.0066824 | 0.9534495 | 1.0628874 | 0.8101203 |
| RBM15 | 2.2573557 | 1.2796771 | 3.981985 | 0.0049305 |
| METTL14 | 1.0803614 | 0.6490056 | 1.798414 | 0.7662529 |
| YTHDC2 | 1.2086098 | 0.7800289 | 1.8726712 | 0.3964114 |
| METTL3 | 1.3247445 | 0.9034581 | 1.9424787 | 0.1498472 |
| ZC3H13 | 0.8419329 | 0.6450645 | 1.0988839 | 0.205483 |
| WTAP | 1.0142761 | 0.8500424 | 1.2102407 | 0.8750236 |
| YTHDF1 | 1.0853404 | 0.974562 | 1.2087109 | 0.1359947 |
| YTHDC1 | 1.0465534 | 0.7931222 | 1.3809652 | 0.7477296 |
| FTO | 1.1024725 | 0.6767262 | 1.7960669 | 0.6952221 |
| YTHDF2 | 1.0876187 | 0.9525856 | 1.2417935 | 0.2143153 |
| ALKBH5 | 1.0056752 | 0.9369585 | 1.0794316 | 0.8754681 |
| LRPPRC | 1.0623019 | 0.9847625 | 1.1459467 | 0.1180766 |
