# Supplementary material for: The significance of m6A RNA methylation regulators in predicting the prognosis and clinical course of HBV-related hepatocellular carcinoma
Source: Mol Med. 2020 Jun 17;26:60. doi: 10.1186/s10020-020-00185-z (PMC7302147; doi:10.1186/s10020-020-00185-z)
Supplement: Supplementary file 3 — Additional file 3: Table S3. The coefficient of the identified genes. [file 10020_2020_185_MOESM3_ESM.docx]

| Table S3. The coefficient of the  identified genes. | |
| --- | --- |
| Gene | Coefficient |
| HNRNPA2B1 | 0.012536274 |
| RBM15 | 0.511744531 |
