# Supplementary material for: The significance of m6A RNA methylation regulators in predicting the prognosis and clinical course of HBV-related hepatocellular carcinoma
Source: Mol Med. 2020 Jun 17;26:60. doi: 10.1186/s10020-020-00185-z (PMC7302147; doi:10.1186/s10020-020-00185-z)
Supplement: Supplementary file 4 — Additional file 4: Table S4. Comparison of the riskScore model with stage, gender, age, and grade models. [file 10020_2020_185_MOESM4_ESM.docx]

| Tabel S4. Comparison of the riskScore model with stage, gender, age, and grade models. | | | |
| --- | --- | --- | --- |
| Models | AUC | Z value | P value |
| risk score | 0.719 | - | - |
| TNM stage | 0.625 | - | - |
| gender | 0.498 | - | - |
| age | 0.692 | - | - |
| grade | 0.56 | - | - |
| risk score VS TNM stage | - | -1.0774 | 0.2813 |
| risk score VS gender | - | 3.0961 | 0.001961 |
| risk score VS age | - | 1.6815 | 0.09267 |
| risk score VS grade | - | 1.5566 | 0.1196 |
