# Supplementary material for: The significance of m6A RNA methylation regulators in predicting the prognosis and clinical course of HBV-related hepatocellular carcinoma
Source: Mol Med. 2020 Jun 17;26:60. doi: 10.1186/s10020-020-00185-z (PMC7302147; doi:10.1186/s10020-020-00185-z)
Supplement: Supplementary file 5 — Additional file 5: Table S5. Identification of the independent prognostic role of prognostic gene signature by univariate analyses using the Cox regression model. [file 10020_2020_185_MOESM5_ESM.docx]

| Table S5. Identification of the independent prognostic role of prognostic gene signature by univariate analyses using the Cox regression model. | | | | |
| --- | --- | --- | --- | --- |
| id | HR | HR.95L | HR.95H | pvalue |
| age | 1.0288641 | 0.986014 | 1.0735764 | 0.1898463 |
| gender | 0.831071 | 0.2344513 | 2.945938 | 0.7744253 |
| stage | 2.0927764 | 1.1863041 | 3.6918974 | 0.0107771 |
| grade | 1.542998 | 0.786001 | 3.029058 | 0.2075683 |
| alcohol | 0.4813654 | 0.10999 | 2.1066709 | 0.3316971 |
| fetoprotein | 0.9999712 | 0.9998857 | 1.0000567 | 0.5088577 |
| new_tumor | 2.509731 | 0.8848272 | 7.1186208 | 0.0836441 |
| riskRcore | 1.0981288 | 1.0280742 | 1.1729571 | 0.005383 |
