# Supplementary material for: The significance of m6A RNA methylation regulators in predicting the prognosis and clinical course of HBV-related hepatocellular carcinoma
Source: Mol Med. 2020 Jun 17;26:60. doi: 10.1186/s10020-020-00185-z (PMC7302147; doi:10.1186/s10020-020-00185-z)
Supplement: Supplementary file 6 — Additional file 6: Table S6. Identification of the independent prognostic role of prognostic gene signature by multivariate analyses using the Cox regression model. [file 10020_2020_185_MOESM6_ESM.docx]

| Table S6. Identification of the independent prognostic role of prognostic gene signature by multivariate analyses using the Cox regression model. | | | | |
| --- | --- | --- | --- | --- |
| id | HR | HR.95L | HR.95H | pvalue |
| age | 1.0198578 | 0.9799697 | 1.0613694 | 0.3340587 |
| gender | 0.8935436 | 0.1979189 | 4.0340774 | 0.8836372 |
| stage | 1.9139416 | 0.9966426 | 3.6755125 | 0.0511925 |
| grade | 1.5559062 | 0.7287469 | 3.3219274 | 0.2533302 |
| alcohol | 0.4560629 | 0.0997874 | 2.0843654 | 0.3112261 |
| riskScore | 1.0867982 | 1.007436 | 1.1724123 | 0.0314403 |
