# Supplementary material for: The significance of m6A RNA methylation regulators in predicting the prognosis and clinical course of HBV-related hepatocellular carcinoma
Source: Mol Med. 2020 Jun 17;26:60. doi: 10.1186/s10020-020-00185-z (PMC7302147; doi:10.1186/s10020-020-00185-z)
Supplement: Supplementary file 7 — Additional file 7: Table S7. The enriched genes in KEGG pathways. [file 10020_2020_185_MOESM7_ESM.docx]

Table S7. The enriched genes in KEGG pathways.

| **Gene** | **KEGG Enrichment** | **RUNNING ES** | **Ranking** |
| --- | --- | --- | --- |
| RPA1 | KEGG_NUCLEOTIDE_EXCISION_REPAIR | -0.6978026 | No.1 |
|  | KEGG_MISMATCH_REPAIR | -0.76484305 | No.1 |
|  | KEGG_DNA_REPLICATION | -0.754055 | No.2 |
| SF3B1 | KEGG_SPLICEOSOME | -0.67515373 | No.1 |
| ZBTB17 | KEGG_CELL_CYCLE | -0.65482366 | No.1 |
| ADA | KEGG_PURINE_METABOLISM | -0.5016493 | No.1 |
